# An evolving perspective on the *Pseudomonas aeruginosa* orphan quorum sensing regulator QscR

**DOI:** 10.3389/fcimb.2014.00152

**Published:** 2014-10-28

**Authors:** Sudha Chugani, Everett P. Greenberg

**Affiliations:** Department of Microbiology, University of WashingtonSeattle, WA, USA

**Keywords:** gene activation, cell-cell signaling, sociomicrobiology, acylhomoserine lactone, bacterial communication

## Abstract

Many *Proteobacteria* govern responses to changes in cell density by using acyl-homoserine lactone (AHL) quorum-sensing (QS) signaling. Similar to the LuxI-LuxR system described in *Vibrio fischeri*, a minimal AHL QS circuit comprises a pair of genes, a *luxI*-type synthase gene encoding an enzyme that synthesizes an AHL and a *luxR-type* AHL-responsive transcription regulator gene. In most bacteria that utilize AHL QS, cognate *luxI* and *luxR* homologs are found in proximity to each other on the chromosome. However, a number of recent reports have identified *luxR* homologs that are not linked to *luxI* homologs; in some cases *luxR* homologs have been identified in bacteria that have no *luxI* homologs. A *luxR* homolog without a linked *luxI* homologs is termed an orphan or solo. One of the first reports of an orphan was on QscR in *Pseudomonas aeruginosa*. The *qscR* gene was revealed by whole genome sequencing and has been studied in some detail. *P. aeruginosa* encodes two AHL synthases and three AHL responsive receptors, LasI-LasR form a cognate synthase-receptor pair as do RhlI-RhlR. QscR lacks a linked synthase and responds to the LasI-generated AHL. QS regulation of gene expression in *P. aeruginosa* employs multiple signals and occurs in the context of other interconnected regulatory circuits that control diverse physiological functions. QscR affects virulence of *P. aeruginosa*, and although it shows sensitivity to the LasI-generated AHL, 3-oxo-dodecanoylhomoserine lactone, it's specificity is relaxed compared to LasR and can respond equally well to several AHLs. QscR controls a set of genes that overlaps the set regulated by LasR. QscR is comparatively easy to purify and study *in vitro*, and has become a model for understanding the biochemistry of LuxR homologs. In fact there is a crystal structure of QscR bound to the LasI-generated AHL. Here, we review the current state of research concerning QscR and highlight recent advances in our understanding of its structure and biochemistry.

## The *pseudomonas aeruginosa* quorum sensing circuit

Quorum sensing (QS) is a cell-to-cell signaling mechanism that allows microbes to adjust their cooperation strategies according to local cell densities. Often QS systems operate by the extracellular release, dissemination, and uptake of acyl-homoserine lactone (AHL) molecules which, upon attaining a threshold concentration affect the activation or repression of target genes. QS systems have been identified in a wide variety of bacterial species where they allow cells to act cooperatively in varied behaviors such as pathogenesis, accessing nutrients, and organizing in groups as biofilms (Fuqua et al., 1996; Miller and Bassler, 2001).

One of the most intensively studied QS systems is that of *Pseudomonas aeruginosa*, a Gram-negative bacterium found in terrestrial and aquatic environments and as an opportunistic pathogen of a range of eukaryotes. *P. aeruginosa* causes infections among immune-compromised humans and in patients with burn wounds or with the genetic disorder cystic fibrosis, where it establishes chronic and stubborn infections. QS appears to play a vital role in the biology and pathogenesis of *P. aeruginosa*. The QS regulatory circuit in *P. aeruginosa* comprises two sub-circuits, Las and Rhl, which utilize the N-acyl homoserine lactone signal molecules 3OC12-HSL and C4-HSL, respectively (Passador et al., 1993; Ochsner et al., 1994; Pearson et al., 1994, 1995; Ochsner and Reiser, 1995). Each circuit includes an AHL signal synthase gene (*lasI* or *rhlI*), and another gene (*lasR* or *rhlR*) encoding a cognate receptor, which regulates target gene expression. The hierarchically dominant Las system positively regulates the Rhl system (Latifi et al., 1996; Pesci et al., 1997). Transcriptome studies suggest that over 300 genes—between 6 and 10% of the genome—are under QS control (Hentzer et al., 2003; Schuster et al., 2003; Wagner et al., 2003). In particular, the Las and Rhl systems are known to regulate the production of multiple extracellular virulence factors including elastase, alkaline protease, pyocyanin, and hydrogen cyanide (Passador et al., 1993; Brint and Ohman, 1995; Van Delden and Iglewski, 1998). Sequencing of the *P. aeruginosa* genome revealed the presence of a third LuxR homolog encoded by the ORF PA1898 (Stover et al., 2000). Unlike LasR and RhlR this homolog, later termed QscR, does not have an associated synthase gene.

## A LuxR-type regulator without a linked LuxI-type synthase

**Figure 1:**
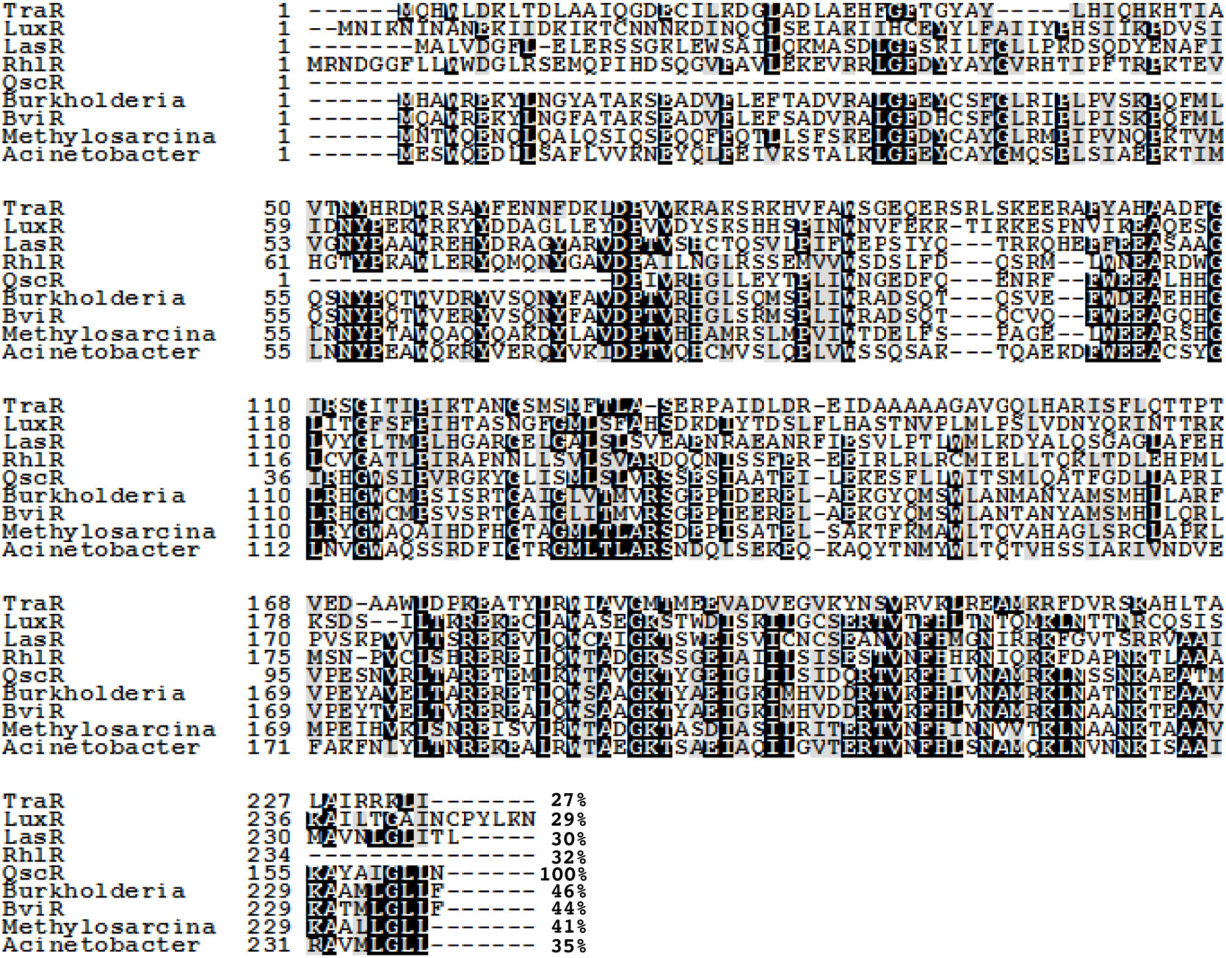
**An alignment of the QscR sequence with previously characterized LuxR homologs TraR, LuxR, RhlR, LasR, and BviR**. We have also included sequences of three ORFs annotated as LuxR-family transcriptional regulators showing significant identity to QscR; *Methylosarcina lacus* (41% identity), *Burkholderia ambifaria* (46% identity), and *Acinetobacter baumannii* (35% identity). Conserved amino acids are shaded in black. Gray shading indicates that 100% of the residues are similar at that position. The numbers at the end of each sequence indicate the percent identity with QscR. The alignment was generated by using the MUSCLE multiple sequence alignment program and the degree of residue shading was determined by using Boxshade. The sequences used in the alignment and their GenBank or NCBI Reference Sequence (RefSeq) accession numbers are *Agrobacterium tumefaciens* TraR (RefSeq: YP_001967610.1), *V. fischeri* LuxR (GenBank: M96844), *P. aeruginosa* LasR (GenBank: M59425), *P. aeruginosa* RhlR (GenBank: L08962), *Burkholderia cepacia* BviR(GenBank: AAK35156.1), *Burkholderia ambifaria* (RefSeq: WP_006749592.1), *Methylosarcina lacus* (RefSeq: WP_024298126.1), and *Acinetobacter baumannii* (GenBank: EXS59053.1).

Sequence alignment shows that QscR has a conserved amino-terminal AHL-binding domain and a conserved carboxy-terminal DNA-binding domain typical of the LuxR family of proteins (Figure 1) (Chugani et al., 2001). QscR is most closely related to BviR from *Burkholderia vietnamiensis* and an uncharacterized LuxR homolog from a methane oxidizing bacterium (Figure 1). QscR is not particularly closely related to LasR, RhlR, or to other orphan LuxR homologs.

Initial genetic studies of QscR suggested that this protein functions to modulate the activity of the Las and Rhl regulons. Both 3O-C12-HSL and C4-HSL accumulated to higher levels earlier in cultures of a *qscR* mutant compared to the wild type. Consistent with this finding, *lasI* and *rhlI* were both prematurely transcribed in the *qscR* mutant. Additional quorum-sensing-controlled genes representative of the Las and Rhl regulons were also expressed early and more strongly in the *qscR* mutant. Thus, QscR appeared to modulate the dynamics of the existing regulatory network by transiently repressing quorum controlled genes early in the growth phase. Cultures of the *qscR* deletion mutant overproduced the virulence factor pyocyanin, and production was evident at a lower culture density than wild type PAO1. The mutant was hypervirulent in the fruit fly *Drosophila melanogaster* infection model(Chugani et al., 2001).

## QscR responds to multiple AHLs

**Figure 2:**
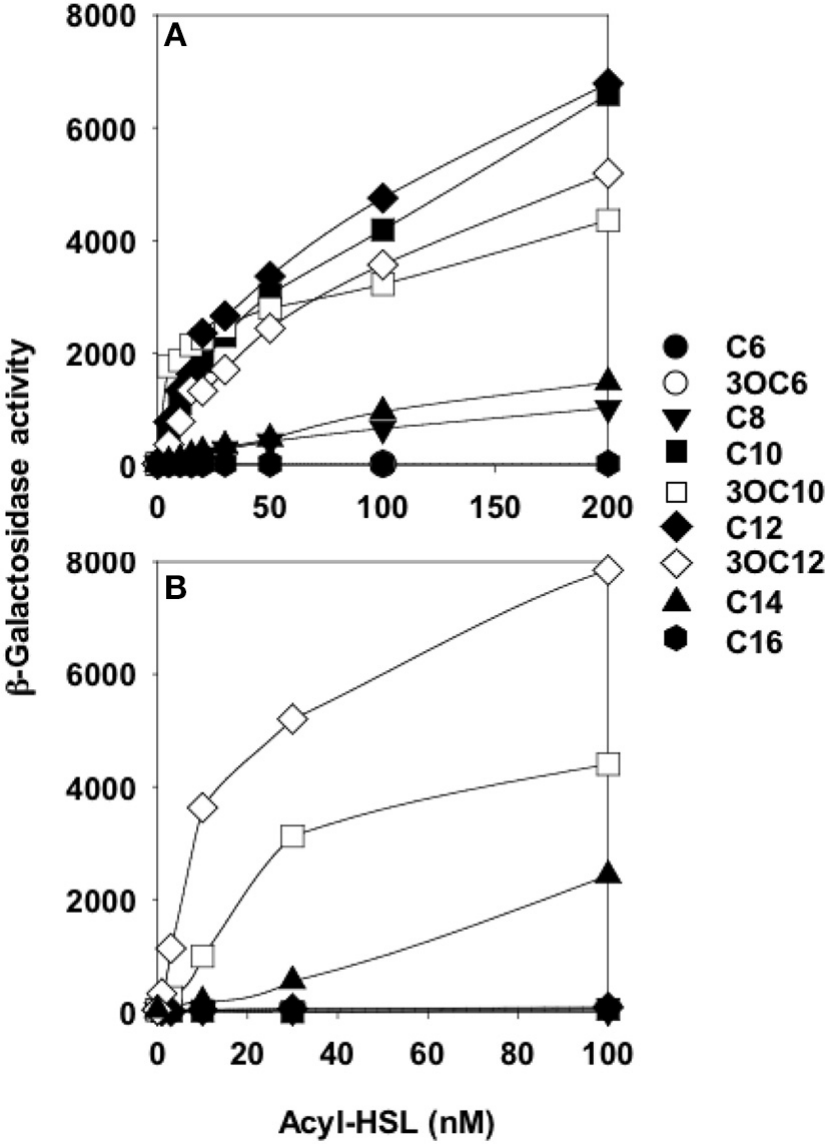
**QscR, unlike LasR, responds to multiple AHLs**. Shown are *lacZ* reporter expression levels for **(A)** *E. coli* containing a *qscR* expression vector and the PA1897–*lacZ* reporter and **(B)** *E. coli* containing a *lasR* expression vector and the *lasI*–*lacZ* reporter. The acyl chains of each AHL are indicated on the right from Lee et al. (2006).

Preceding studies with LuxR homologs had shown that purification of active, soluble protein required the presence of the cognate AHL during growth (Zhu and Winans, 1999; Schuster et al., 2004; Urbanowski et al., 2004). Overproduction of His-tagged QscR in a *P. aeruginosa* strain producing 3OC12-HSL yielded soluble QscR (Lee et al., 2006). There was a direct correlation between the soluble fraction and the presence of 3OC12-HSL but not C4-HSL, the second *P. aeruginosa* AHL. The *qscR* gene is divergently transcribed from PA1897, which codes for a hypothetical protein, and the *qscR*-PA1897 intergenic region includes an inverted repeat showing similarity to known AHL binding sites. DNase I footprinting analysis with purified QscR and added 3OC12-HSL identified this inverted repeat as a bona fide QscR binding site. This was further confirmed by gel-shift experiments, which also showed that binding of QscR was cooperative. This study seemed to suggest that the *in vitro* DNA-binding activity of QscR was strongly dependent upon exogenously added 3OC12-HSL. Experiments using a transcriptional reporter in *Escherichia coli* confirmed that PA1897 was directly activated by QscR-3OC12-HSL. An *in silico* search of the PAO1 genome identified a second gene, PA5351 that encodes rubredoxin 1, that had a putative QscR binding site in its promoter. Although QscR-3OC12-HSL can bind to this site, the binding was not cooperative and the apparent binding affinity was lower than the affinity for the PA1897 binding site. A limited *in vitro* analysis of the promoters of PA1897 and selected LasR-regulated genes revealed that despite significant similarity between the binding sites and use of a common ligand by the two transcription factors, QscR is unable to activate LasR-dependent genes and vice versa. Thus, although QscR appears to transiently repress a subset of LasR and RhlR regulated genes, this regulation appears to be largely indirect and the mechanism by which this occurs remains unclear. A particularly intriguing finding was that QscR has a broad signal specificity. It can activate a PA1897*–lacZ* reporter at nM concentrations of C8, C10, 3OC10, C12, 3OC12, and C14-HSLs (Figure 2). This suggests that in certain mixed speices populations QscR may integrate multispecies signaling by responding to AHLs produced by other bacteria.

In an *in vivo* chemical cross-linking analysis using *E.coli* producing QscR with either LasR or RhlR in the absence of AHL, Ledgham et al. (2003) identified apparent QscR-LasR and QscR-RhlR heterodimers. Based on fluorescence anisotropy analysis of the *E. coli* cells, it was proposed that QscR exists as an oligomer in *E. coli* and that it is destabilized by addition of either 3O-C12-HSL or C4-HSL. This work added to the possible explanations for the QscR-mediated delays in expression of several LasR- and RhlR-activated genes. QscR might directly interact with LasR- or RhlR-controlled promoters, QscR might bind to 3OC12-HSL, the LasR signal, or C4-HSL, the RhlR signal, or QscR might form inactive heterodimers with LasR or RhlR.

## The QscR regulon includes genes that are directly and indirectly regulated

For a more comprehensive analysis of the QscR regulon, the transcriptomes of the *qscR* mutant and PAO1 were compared at several points during growth by using microarrays (Lequette et al., 2006). This study identified 424 genes that were QscR-controlled. Among these, 76 genes were induced and a majority (329 genes) were repressed by QscR. It was conceivable that a subset of genes, particularly repressed genes, was regulated indirectly, perhaps through the formation of heterodimers with LasR or RhlR or through signal sequestration; mechanisms independent of the ability of QscR to bind DNA. In contrast, genes that were activated by QscR and genes that were not part of the LasR or RhlR regulons were more likely directly regulated by QscR and dependent on its DNA binding activity. To identify this set of genes, *P. aeruginosa qscR* mutants containing an inducible chromosomal copy of either full-length *qscR* or a *qscR* allele lacking the DNA-binding domain were transcriptionally profiled under inducing conditions. A set of 38 QscR-regulated genes identified in the previous microarray experiment also required the QscR DNA-binding domain. Some of these genes were activated and some were repressed by elevated expression of full-length *qscR*.

## Biochemical analysis of QscR suggests a new model for LuxR homologs

A biochemical analysis of purified native QscR provided unexpected insights into the properties of QscR that pointed to a new model for LuxR homologs in their free and signal-bound states (Oinuma and Greenberg, 2011). As observed in a previous study using His-tagged QscR (Lee et al., 2006), *E. coli* grown in the presence of 3OC12-HSL yielded higher levels of soluble native QscR compared to cells grown without added 3OC12-HSL. QscR seemed to retain 3OC12-HSL throughout the purification process and was therefore able to bind target DNA in the absence of added 3OC12-HSL. However, DNA binding was significantly stimulated by addition of 3OC12-HSL to the reaction buffer. These seemingly curious findings can be explained by the equilibrium binding dynamics of QscR and 3OC12-HSL. Dilution of purified QscR-3OC12-HSL into DNA-binding buffer likely causes a shift in equilibrium toward the ligand-free form of QscR and a release of 3OC12-HSL. As expected then, addition of 3OC12-HSL to the reaction stimulates DNA binding.

As shown previously with His-tagged QscR (Lee et al., 2006), gel filtration experiments with native QscR showed that at a low concentration it exists as a monomer in solution. However, at higher concentrations QscR seemed to dimerize in a concentration dependent manner.

Although previous work showed that 3OC6-HSL was not able to activate transcription of the PA1897-*lacZ* reporter fusion in recombinant *E. coli* (Lee et al., 2006), the presence of 3OC6-HSL did facilitate production of soluble QscR. Experiments with QscR purified from *E.coli* grown in the presence of 3OC6-HSL indicated that ligand-free QscR is unstable and that acyl-HSL binding seems to stabilize the protein. Evidence exists in favor of the idea that other LuxR homologs may also be stabilized by acyl-HSLs both *in vitro* and *in vivo* (Zhu and Winans, 1999, 2001). Purification of several other LuxR homologs in the soluble form seems to be contingent upon the presence of their cognate acyl-HSLs. The TraR protein in *Agrobacterium tumefaciens* is targeted for proteolysis in the absence of its ligand 3OC8-HSL (Zhu and Winans, 2001). In fact, studies with TraR supported a view that LuxR family members may be unable to fold into an active polypeptide in the absence of an appropriate AHL.

**Figure 3:**
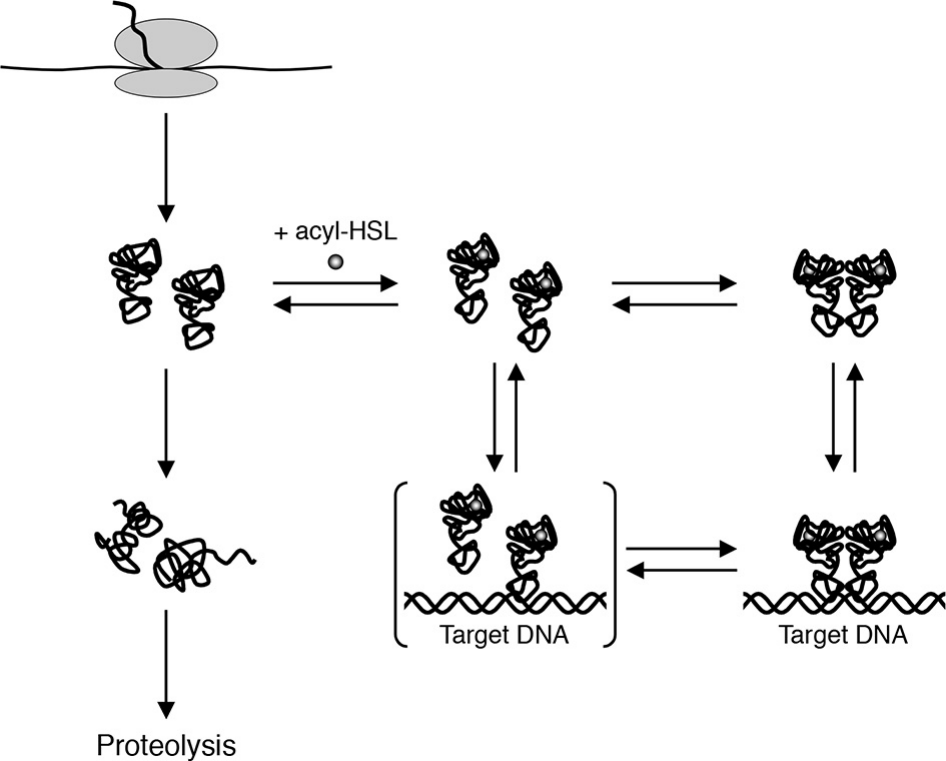
**A model for biochemical states of QscR *in vivo***. Nascent QscR polypeptides can fold into active forms and exist as monomers at low concentrations. The monomers can either refold into inactive forms that are subsequently proteolyzed or they can bind AHLs and form homodimers capable of binding target DNA from Oinuma and Greenberg (2011).

Experiments done with purified QscR-3OC6-HSL point to an alternate model for LuxR homolog states *in vivo*. Reporter gene fusion experiments showed that QscR-3OC6-HSL is able to activate transcription only slightly. Purified QscR-3OC6-HSL remained soluble although it could bind target DNA only at high concentrations of the AHL. Further experiments showed that QscR binds 3OC6-HSL with a low affinity and that it was possible to replace 3OC6-HSL with other AHL ligands before QscR aggregated into an inactive form. For example, QscR that had dissociated from 3OC6-HSL upon dilution could bind target DNA if 3OC12-HSL was present in the reaction buffer. The fact that QscR could disengage from 3OC6-HSL and bind DNA in the presence of another AHL questioned the prevailing view that in order to maintain a functional conformation, nascent polypeptides of LuxR homologs are required to fold around their cognate AHLs (Zhu and Winans, 1999, 2001; Schuster et al., 2004). A more likely model, which was consistent with the new findings, proposed that the nascent polypeptides fold into functional monomers that are relatively unstable (Figure 3). The monomers can bind their cognate AHLs and can form homodimers that bind DNA targets with a high affinity. However, in the absence of suitable AHLs the protein returns to its monomeric state and the monomers either refold into non-functional aggregates or are subject to proteolysis.

The availability of soluble QscR through purification in presence of 3OC6-HSL also made assays for activity and binding dynamics in presence of other AHLs feasible. Purified QscR-3OC6-HSL was used to estimate binding affinities of QscR for other AHLs and for target DNA. Results from gel-shift DNA-binding experiments showed that QscR has a similar binding affinity for 3OC12-, C12-, and C10-HSLs. The affinity for 3OC10-HSL is lower than for the other AHLs. For all tested AHLs, binding of QscR to target DNA appeared to be cooperative (Oinuma and Greenberg, 2011).

## Crystal structure of QscR-3OC12-HSL

**Figure 4:**
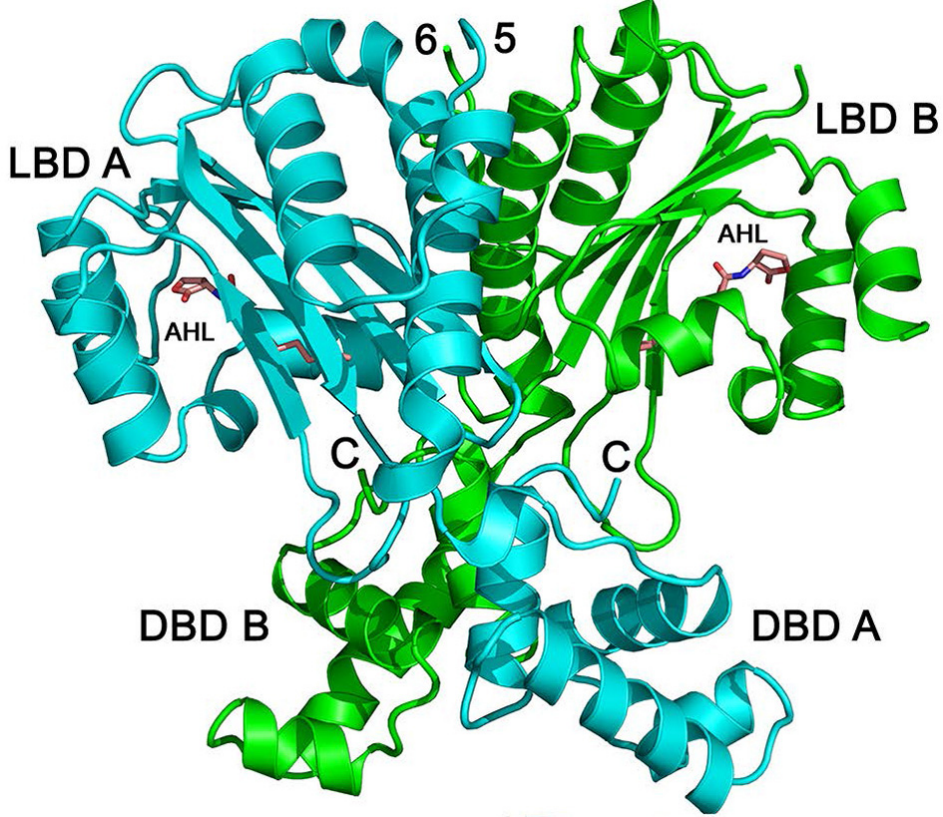
**Structure of QscR bound to 3OC12-HSL**. The QscR monomers are shown in cyan and green and 3OC12-HSL is shown in orange. The ligand-binding domain (LBD) and the DNA- binding domain (DBD) are also indicated for each chain from Lintz et al. (2011).

Detailed biochemical and structural studies of LuxR homologs have often been hampered due to protein instability in absence of ligand or protein insolubility at high concentrations. The biochemical characterization of QscR provided a basis for obtaining a substantial amount of soluble and stable QscR. It was thus feasible to determine the crystal structure of full-length QscR when bound to 3OC12-HSL (Figure 4). Previously, structures of full-lengthTraR (*Agrobacterium tumefaciens*) bound to 3OC8-HSL and target DNA, full-length CviR (*Chromobacterium violaceum*) bound to antagonist, and the N-terminal AHL-binding domains of LasR (*P. aeruginosa*) and SdiA (*E. coli*) had been reported (Vannini et al., 2002; Zhang et al., 2002; Yao et al., 2006; Bottomley et al., 2007; Chen et al., 2011). The crystal structure of QscR revealed that the dimer bound to 3OC12-HSL has some shared and some unique features when compared with other available LuxR structures (Lintz et al., 2011). A feature shared with CviR but not with TraR, is the almost symmetric cross-subunit conformation of the QscR homodimer that allows for an extensive dimerization interface. Mutational analyses of QscR confirmed the *in vivo* functional relevance of the interface and suggested that dimerization is important for the response of QscR to AHLs. Further, modeling of QscR-DNA interactions suggests that the DNA-binding domains in the cross-subunit conformation are poised to bind DNA. Because the CviR structure was determined with bound antagonists and not AHLs, the generalized relevance of the cross-subunit architecture is unclear. The results with QscR seem to suggest that this structural similarity may be a more generalized feature of LuxR homologs.

As expected of two LuxR-type proteins that recognize the same ligand, the binding pocket surface areas, packing densities, and pocket volumes are almost identical in QscR and LasR. As noted earlier, QscR has a relaxed specificity for AHLs compared to LasR (Lee et al., 2006). A comparison with equivalent residues in other known LuxR structures suggested that Ser56 of QscR influences its specificity for AHLs allowing both 3-oxo-substituted and unsubstituted AHLs to bind. It was proposed that differences in the interactions involving the 3-oxo-position of the acyl chain may form the basis of the relaxed signal specificity relative to LasR.

The ligand-binding domains of 3OC12-HSL-bound QscR and LasR also show a conserved “internalized” conformation of the AHL in the binding pocket whereby the acyl chain of 3OC12-HSL is similarly embedded in a cavity near the region that forms the ligand-binding and the DNA-binding domain interface in QscR (Bottomley et al., 2007; Zou and Nair, 2009). This conformation, which influences the interactions between the ligand-binding and the DNA-binding domains is not observed in AHLs or antagonists with short acyl chains. Thus, it is likely that the acyl-chain length plays a role in the mode of AHL binding. In contrast to other LuxR-type proteins, homodimers of both QscR and LasR have almost identical interactions between the AHL-binding domains. Taken together, the data suggest that the conformation of the QscR and LasR-bound AHL likely promotes dimerization and subsequent binding of the dimer to DNA through an allosteric mechanism.

A structural comparison of 3OC12-HSL-bound QscR with TraR-3OC8-HSL-DNA revealed some interesting differences. While QscR-3OC12-HSL is nearly symmetric, the subunit architecture of TraR-3OC8-HSL bound to DNA is asymmetric and unlike QscR, shows fewer interactions between the ligand-binding and DNA-binding domains. Evidence from structural analyses of TraR bound to its antiactivator TraM suggests that ligand-bound TraR is also unlikely to adopt the cross-subunit architecture observed with QscR. The linker between the ligand-binding and the DNA-binding domains may have evolved for distinct functions in QscR and TraR. In QscR and perhaps LasR, the linker functions to allow extensive contacts with the ligand-binding and DNA-binding domains whereas in case of TraR, the linker probably evolved to bind TraM and not necessarily to interact with the ligand-binding and DNA-binding domains. Thus, the differences in architectures of full-length LuxR proteins may be reflective of differences in their physiological activities and interactions with other regulatory factors.

While the structure of CviR bound to antagonists resembled QscR-3OC12-HSL in the near symmetry of the cross-subunit architecture, unlike QscR, which is poised to bind DNA, the DNA-binding domains of CviR are in a conformation that prevents DNA binding. The structural studies suggest that this architecture would allow conversion of CviR from the inactive form to a form that resembles QscR through only a small configurational alteration that permits interaction between the C-terminal dimerization helices. Further studies will be required to determine whether this configuration persists in active forms of the proteins when bound to target DNA.

A few groups have used a biochemical approach to generate tools that could aid in the study of QscR. Liu et al. (2010) used *in vitro* and *in vivo* assays to identify several furanones that inhibit 3OC12-HSL-dependent activation of QscR. In another study, Mattmann et al. (2011) designed several highly active QscR-selective agonists and antagonists. This study also found that the most potent antagonists for QscR had sterically bulky acyl chains (for example, N-benzoyl acyl groups) and that agonists had branched lipophilic acyl groups. More recently, Weng et al. (2014) reported that a synthetic ligand with an open ring structure, N-decanoyl-l-homoserine benzyl ester, was able to activate QscR. This work suggested that the acyl chain may be more critical for ligand binding to QscR than the lactone ring.

## Conclusions

*P. aeruginosa* QS is one of the best understood cell-cell signaling systems in bacteria. It has two synthase and receptor pairs that allow it to respond to self-generated AHL signals and a third orphan receptor with no cognate synthase. In this review we have summarized advances in our understanding of the *P. aeruginosa* orphan or solo QS receptor QscR. Although much has been learned, especially about the biochemistry and structure of QscR, many questions still remain unanswered. While it is clear that QscR influences the expression of genes controlled by both the Las and the Rhl systems, the precise mechanism by which this is affected remains unclear. Bacterial populations in nature exist as dynamic and often polymicrobial communities. QscR responds not only to the *P. aeruginosa* signal 3OC12-HSL but also to multiple other AHLs. It thus has the potential to significantly widen the scope of QS regulation in *P. aeruginosa* through integration of signals produced by other cohabiting species. Although intriguing, the complex nature of microbial growth in mixed communities makes it challenging to test this possibility under laboratory conditions.

A complex regulatory network such as the *P. aeruginosa* QS system operates on a global scale. It controls the expression of numerous genes and is in turn also subject to regulation by other modulators. For example, *lasR* transcription is modulated by Vfr and RsaL (Fagerlind et al., 2003; Viretta and Fussenegger, 2004; Ward et al., 2004). Liang et al. (2012) showed that VqsR binds to the *qscR* promoter region and negatively regulates *qscR*. It is likely that future studies will identify additional modulators of QscR activity or synthesis. It is also important to note that although QS controlled genes can also be regulated at a translational or post-transcriptional level, much of the available information on QscR and *P. aeruginosa* QS is based on transcriptome analyses. Another level of regulation of QS systems involving sRNAs has been described in *Vibrio fischeri* and *Vibrio harveyi* (Lenz et al., 2004; Tu and Bassler, 2007). The possibility of similar regulatory mechanisms in *P. aeruginosa* will have to be considered as well.

QscR has been characterized in great detail by biochemical methods and through structural analysis. These studies have revealed a wealth of information and established a framework for further understanding of not only QscR but other LuxR homologs as well. Because QscR and LasR respond to the same ligand, recent reports of QscR-specific modulators including small molecules and synthetic AHLs provide a means to selectively understand the role of QscR within the *P. aeruginosa* QS system. This approach could help address the larger question regarding the utility of a QS circuit that includes two signal receptors with a shared ligand.

### Conflict of interest statement

The authors declare that the research was conducted in the absence of any commercial or financial relationships that could be construed as a potential conflict of interest.
