# Corrigendum: An evolving perspective on the *Pseudomonas aeruginosa* orphan quorum sensing regulator QscR

**DOI:** 10.3389/fcimb.2014.00181

**Published:** 2015-01-28

**Authors:** Sudha Chugani, Everett P. Greenberg

**Affiliations:** Department of Microbiology, University of WashingtonSeattle, WA, USA

**Keywords:** bacterial communication, cell-cell signaling, transcription factors, sociomicrobiology, gene expression regulation

Figure 1 of the article by Chugani and Greenberg contains errors in the QscR sequence used in the alignment, which we hereby rectify. In the original figure the N-terminal portion of QscR shows as dashes. We resubmit Figure 1 with corrections in the sequence.

**Figure 1:**
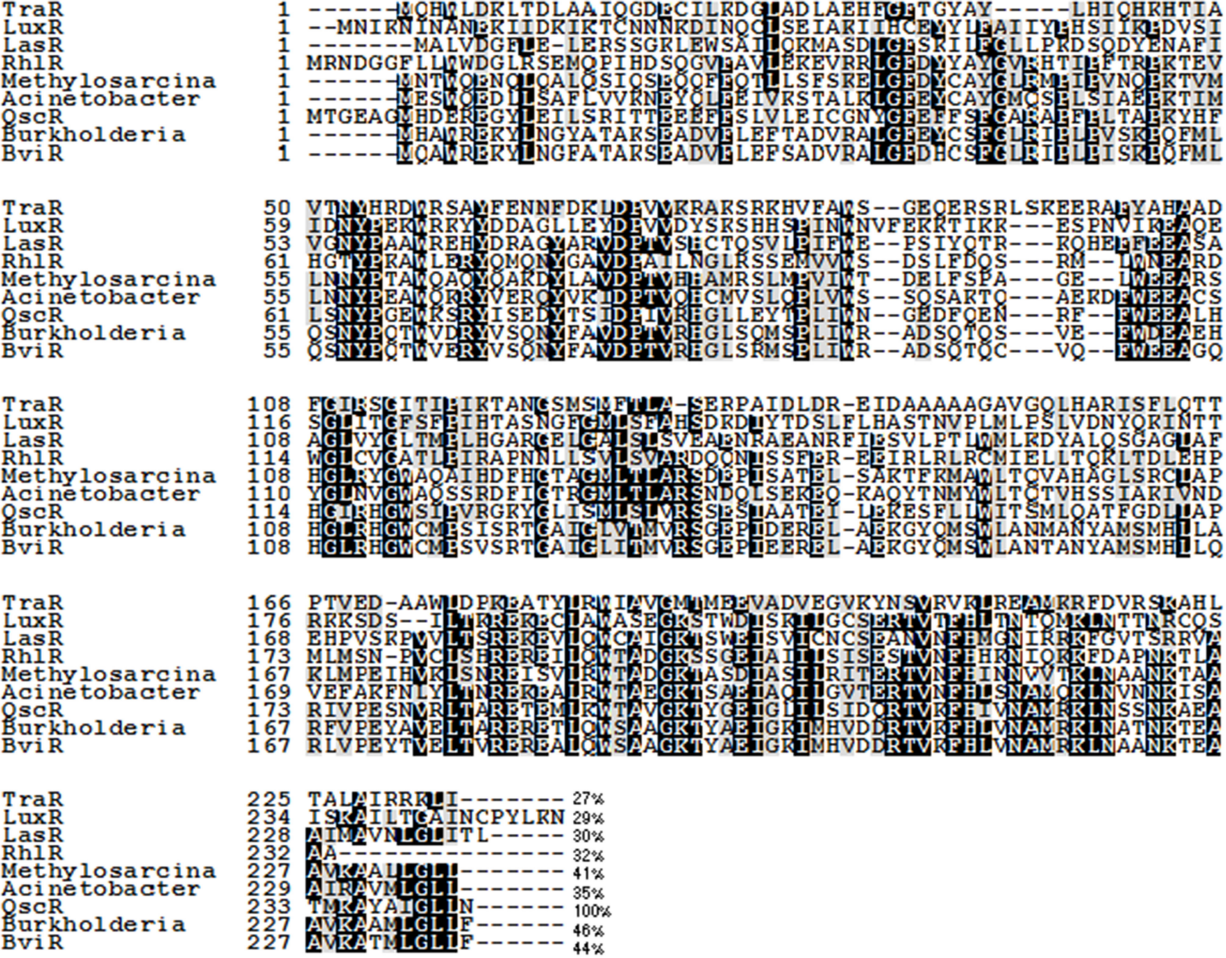
**An alignment of the QscR sequence with previously characterized LuxR homologs TraR, LuxR, RhlR, LasR, and BviR**. We have also included sequences of three ORFs annotated as LuxR-family transcriptional regulators showing significant identity to QscR; *Methylosarcina lacus* (41% identity), *Burkholderia ambifaria* (46% identity), and *Acinetobacter baumannii* (35% identity). Conserved amino acids are shaded in black. Gray shading indicates that 100% of the residues are similar at that position. The numbers at the end of each sequence indicate the percent identity with QscR. The alignment was generated by using the MUSCLE multiple sequence alignment program and the degree of residue shading was determined by using Boxshade. The sequences used in the alignment and their GenBank or NCBI Reference Sequence (RefSeq) accession numbers are *Agrobacterium tumefaciens* TraR (RefSeq: YP_001967610.1), *V. fischeri* LuxR (GenBank: M96844), *P. aeruginosa* LasR (GenBank: M59425), *P. aeruginosa* RhlR (GenBank: L08962), *Burkholderia cepacia* BviR (GenBank: AAK35156.1), *Burkholderia ambifaria* (RefSeq: WP_006749592.1), *Methylosarcina lacus* (RefSeq: WP_024298126.1), and *Acinetobacter baumannii* (GenBank: EXS59053.1).

## Conflict of interest statement

The authors declare that the research was conducted in the absence of any commercial or financial relationships that could be construed as a potential conflict of interest.

